# Core-binding factor acute myeloid leukemia: long-term outcome of 70 patients uniformly treated with “7+3”

**DOI:** 10.1038/s41408-022-00654-0

**Published:** 2022-04-07

**Authors:** K. H. Begna, X. Xu, N. Gangat, H. Alkhateeb, M. M. Patnaik, A. Al-Kali, M. A. Elliott, W. J. Hogan, M. R. Litzow, C. C. Hook, A. P. Wolanskyj-Spinner, A. Mangaonkar, R. He, A. Pardanani, M. Shah, R. P. Ketterling, A. Tefferi

**Affiliations:** 1 Department of Internal Medicine and Division of Hematology, Rochester, MN USA; 2 Division of Hematopathology and Department of Laboratory Medicine and Pathology, Mayo Clinic, Rochester, MN USA

**Keywords:** Cancer therapy, Cancer

**TO THE EDITOR:**

Core-binding factor acute myeloid leukemia (CBF-AML) has been associated with relatively better prognosis following intensive AML directed therapy, typically consisting of 7 days of continuous intravenous (IV) cytarabine and 3 days of anthracycline IV push (“7 + 3”), followed by multiple cycles of high-dose cytarabine consolidation [1, 2]. However, such therapy has been associated with a 30–50% relapse rate [3], thus warranting consideration of alternative induction regimens including FLAG-IDA (fludarabine, cytarabine, idarubicin and filgrastim) and induction enhancements with gemtuzumab ozogamicin (GO) [4–7] or dasatinib [8]. Such therapeutic approaches have not been tested in a controlled setting and retrospective comparisons require examination of data from multiple sources that utilized 7 + 3 in large cohorts with long-term follow-up.

After obtaining approval from the Mayo Clinic Institutional Review Board, patients with cytogenetically and/or molecularly (fluorescence in situ hybridization; FISH) confirmed cases of CBF-AML were identified from the Mayo Clinic AML database. Study patients were seen between December 1998 and May 2021 and follow-up information was updated as of June 2021. Study inclusion criteria included i) age >18 years; ii) documentation of newly-diagnosed AML associated with t(8;21)(q22;q22.1), inv(16)(p13.1q22) or t(16;16)(p13.1;q22) karyotype and/or the corresponding FISH identified fusion genes, including *RUNX1/RUNX1T1* and *CBFβ/MYH11;*iii) induction therapy with “7 + 3”. Patients who received other forms of intensive therapy were excluded. Conventional methods were used for cytogenetic and molecular studies, including next-generation sequencing. Statistical analysis was performed using JMP Pro 14.0.0 software package, SAS Institute, Cary, NC.

A total of 90 patients with CBF-AML were identified; 85 (94%) patients received intensive chemotherapy, including cytarabine plus anthracycline (7 + 3; *n* = 70), 7 + 3 + GO (*n* = 3), and other combinations/additions (*n* = 17). The current study is focused on the 70 patients (median age 48 years, range 20–73; males 59%) treated with “7 + 3” induction followed by high dose cytarabine consolidation (Table 1). The cytogenetic/molecular profiles were t(8;21)(q22;q22.1)/*RUNX1/RUNX1T1* and inv(16)(p13.1q22)/t(16;16)(p13.1;q22)/*CBFβ/MYH11* in 31 (44%) and 39 (56%) patients, respectively; the two molecular subgroups were similar in terms of demographics, *KIT* and other pathogenetic mutation status, and treatment remission and relapse rates (Table 1). Fifty-seven (81%) of the 70 study patients presented *de novo* and 13 (19%) were therapy related.

**Table 1:** Clinical Characteristics of CBF AML patients uniformly treated with “7+3”.

Characteristics	Intensive therapy “7 + 3”	*Fusion Subtype*		*P* value
		*MYH11/CBFB n* (%)	*RUNX1T1/RUNX1 n* (%)	
Patients	70 (100)	39 (56)	31 (44)	
Gender:				0.7
Male	41 (59)	22 (56)	19 (61)	
Female	29 (31)	17 (44)	12 (39)	
Age, median (range)	48 (20–73)	50 (20–73)	47 (20–73)	
Age at Diagnosis				0.3
less than 60	50 (71)	30 (77)	20 (65)	
Age ≥60 years	20 (29)	9 (23)	11 (35)	
Alive	39 (56)	22 (56)	17 (55)	0.9
dead	31 (44)	17 (44)	14 (45)	
*Type of AML*				0.3
Primary	57 (81)	30 (77)	27 (87)	
Therapy-Related	13 (19)	9 (23)	4 (13)	
Hgb (*n* = 85) median(range)	8.7 (5–14.5)	8.5 (5–14.5)	9.2 (5–13.8)	
WBC (*n* = 85) median(range)	10.4 (1.7–256)	7 (1.7–42)	30 (2.1–256)	
Platelet (*n* = 85) median(range)	31 (3–361)	34 (7–185)	31 (3–361)	
Blasts (PB) (*n* = 82) median(range)	29.5 (0–85)	22 (2–71)	33 (0–85)	
Blasts (BM) (*n* = 84) median(range)	54 (6–91)	54 (10–91)	54 (6–83)	
Additional Cytogenetics abnormalities	23	9	14	0.1
NGS (*n* = 29)***	29	11	18	
*FLT3-ITD* (27 negative)	3	1	2	
*CEBPA* (21 negative)	1	1	0	
*IDH1* (17 negative)	1	1	0	
*KIT (20 negative)*	8	5	3	0.4
*WT1* (20 negatives)	1	1	0	
*Remission*				0.01
Complete remission (CR)	52 (74)	34 (87%)	18 (58%)	
CRi*	17 (24)	5 (13%)	12 (39%)	
No	1 (1%)	0	1 (1%)	
*Relapse*				0.2
Yes	31 (45)	19 (49)	11 (36)	
No	38 (55)	20 (51)	19 (61)	
*Time to relapse*				0.8
Early (<6 months)	6 (19)	4	2	
Intermediate (6–12 months)	12 (39)	7	5	
Late (>12 months)	13 (42)	9	4	
Allogeneic SCTX**				0.02
Yes	24 (35)	18 (46)	6 (20)	
No	45 (65)	21 (54)	24 (80)	

^*^CRi: CR with incomplete count recovery.

^**^Reasons for Allogeneic Stem cell Transplant (Allogenic SCTX) in CR1: [10] 1 case because of additional myeloid sarcoma; 2 FLT3-ITD positive, 1 additional chromosomal abnormality including trisomy 4, 3 therapy related, 2 KIT mutation and 1 CNS involvement.

^***^NGS (next-generation Sequencing) some genes were testes as stand-alone test with PCR before the availability of NGS testing.

Overall, 98.5% (69 patients) of the patients achieved remission, including 74.3% (52/70) complete remission (CR) and 24.3% (17/70) CR with incomplete count recovery (CRi). Among the 69 patients with CR/CRi, 10 underwent allogeneic hematopoietic stem cell transplant (AHSCT) in CR1 based on suspected high-risk disease, which was considered in the context of therapy-related AML (*n* = 3); extramedullary disease (*n* = 1) or presence of additional chromosome abnormalities (*n* = 1), concomitant *KIT* and CNS involvement (*n* = 3) and *FLT3*-ITD mutations (*n* = 2). An additional 14 patients received AHSCT in CR2. The median (range) of age-appropriate HIDAC was 3 (0–4) in both with and without alloSCT. In those with alloSCT (one zero, three 1, three 2, eight 3 and seven 4 consolidation therapies) and in those without alloSCT (one zero, 5 one, 7 two, 12 three, and 19 four consolidation respectively), and not statistically different. Age-appropriate HIDAC was given to 49/49 (100%) patients (<60 years) and 18/18 (100%) patients of 60 years and above; and in 3 patients with age 73, 63, and 47 # of consolidation unknown. After a median follow-up of 46 months (0.5–231), 31 (45%) relapses and 31 (44%) deaths were documented. One patient, a 62-year-old with therapy-related AML, died before the day 14 bone marrow biopsy with early mortality of 1% (1/70); overall relapse rate was 49% (19/39) in patients with *CBFβ/MYH11* and 36% (11/31) in those with *RUNX1/RUNX1T1* (*p* = 0.2).

**Fig. 1:**
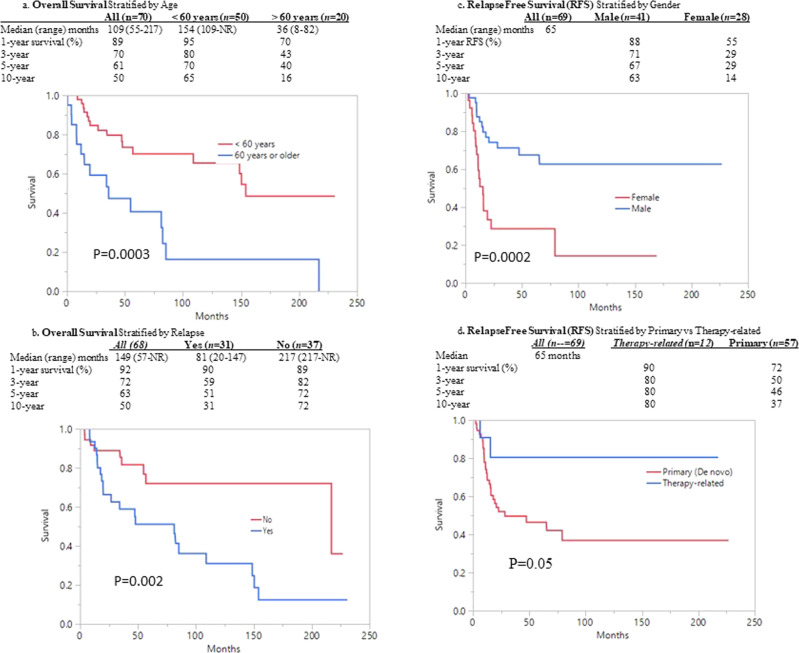
Overall and Relpase Free Survival in 70 patients with Core-Binding factor AML. **a** Overall Survival Stratified by Age; **b** Overall Survival Stratified by Relapse; **c** Relapse free survival stratified by Gender; **d** Relapse free survival stratified by primary vs. therapy-related.

The 1-, 3-, 5- and 10-year overall survival (OS) rates were 90%, 70%, 61% (at risk *n* = 27) and 50% (at risk *n* = 15), respectively. Multivariable analysis identified age ≥60 years (HR 3.5, 95% CI 1.6–7.3) and disease relapse (HR 3.3, 95% CI 1.5–7.4) as the only two risk factors for OS (Fig. 1a, b); accordingly, the 1-, 3-, 5-, and 10-year OS for patients below age 60 years was 95%, 80%, 70% (at risk *n* = 21) and 65% (at risk *n* = 14) respectively. The type of CBF fusion protein (*p* = 0.2), the presence of *KIT* mutation (*n* informative=22; *p* = 0.9) or additional chromosomal abnormalities (*p* = 0.7) did not carry independent predictive value for OS. The 1-, 3-, 5- and 10-year relapse-free survival rates were 75, 55, 52, and 45%, respectively. Multivariable analysis identified the female gender (HR 3.4, 95% CI 1.6–7.1) as the only adverse risk factor for relapse-free survival; primary vs therapy-related CBF-AML tended to be associated with inferior relapse-free survival (HR 3.1, 95% CI 0.7–13.3; *p* = 0.07) (Fig. 1c, d); the higher risk of relapse in primary vs therapy-related disease was not accounted for by differences in utilization of AHSCT or karyotype (Table). None of the patients who received AHSCT in CR1 (*n* = 10) relapsed. Similarly, only 1 of 14 patients transplanted in CR2 relapsed. By contrast, the relapse rate among patients achieving CR/CRi that was not consolidated with AHSCT was 38% (17/45).

The current study confirms the near 100% CR/CRi rate in CBF-AML treated with 7 + 3. The study also underlines the substantial risk of relapse, which was fortunately successfully addressed with AHSCT, regardless of the timing for transplant. Our observations suggest that pre-emptive AHSCT might reduce the risk of relapse; however, deferring AHSCT until after disease relapse did not appear to compromise ultimate outcome. Whether or not newer treatment approaches, such as the addition of GO [5–7] or dasatinib [8] to 7 + 3 or use of alternative induction regimens such as FLAG-IDA are effective in reducing relapse rates in CBF-AML require examination in a controlled setting. Similarly, controlled studies are needed to examine the role of maintenance therapy, including that with hypomethylating agents., especially in the context of minimal residual disease (MRD). Although the number of informative cases was small, our observation suggesting a more favorable outcome in therapy-related CBF AML was unexpected and not consistent with previous reports [9, 10]. Whether or not the observed discrepancy between studies relates to differences in patient demographics, disease biology or treatment regimens is plausible but not certain [11]. The same can be said regarding our observation regarding a higher rate of relapse in women, which was also reported in another study [12].
